# The Association of the Potential Risk Factors and Nutrition Elements with Abortion and Calving Rates of Egyptian Buffaloes (*Bubalus bubalis*)

**DOI:** 10.3390/ani11072043

**Published:** 2021-07-08

**Authors:** Walaa M. Essawi, Ali Ali El-Raghi, Fatma Ali, Mohamed A. Nassan, Ahmed N. F. Neamat-Allah, Mahmoud A. E. Hassan

**Affiliations:** 1 Department of Theriogenology, Faculty of Veterinary Medicine, Aswan University, Aswan 81528, Egypt; walaamohamed1995@yahoo.com; 2 Department of Animal Production, Faculty of Agriculture, Damietta University, Damietta 34517, Egypt; ali21384@yahoo.com; 3 Physiology Department, Faculty of Veterinary Medicine, Aswan University, Aswan 81528, Egypt; fatma_ali@vet.svu.edu.eg; 4 Department of Clinical Laboratory Sciences, Turabah University College, Taif University, P.O. Box 11099, Taif 21944, Saudi Arabia; moh_nassan@yahoo.com; 5 Department of Clinical Pathology, Faculty of Veterinary Medicine, Zagazig University, Zagazig City 44511, Egypt; 6 Animal Production Research Institute (APRI), Agriculture Research Center, Ministry of Agriculture, Dokki, Giza 12619, Egypt; m.hassan55213@gmail.com

**Keywords:** Egyptian buffalo, abortion, calving rate, mineral, blood metabolites

## Abstract

**Simple Summary:**

The association of the risk factors, serum constituents related to abortion and calving rates were investigated. Multiparous buffaloes were less likely to abort and more likely to calving than primiparous. Dry buffaloes had lesser odds of abortion and were six times more likely to give birth than those lactating. Conversely, the sex of the fetus had no impact. In aborted animals, serum glucose and copper were higher, whereas urea, uric acid, total proteins, total cholesterol, phosphorus, magnesium and iron were lower than in normal pregnancy. This emphasizes that risk factors and serum constituents associated with abortion aid to construct suitable preventive measures to raise reproductive performance.

**Abstract:**

The aim of the present study was to investigate risk factors, serum minerals, and metabolites associated with non-infectious abortion and calving rates of Egyptian buffaloes. Data were obtained from 364 pregnant buffaloes of different ages and parities over 7 years from 2014 to 2020. Body condition score (BCS) was a risk factor regarding abortion and calving; the thinnest buffaloes were more likely to abort and less likely to calving than those with body energy reserves. In comparison with the spring season, aborting probability decreased 49.7% the odds ratio (OR = 0.503), while the chance of calving increased 72.1% (OR = 1.721) during winter. The parity was another significant factor related to abortion and calving rates; multiparous buffaloes were less likely to abort and more likely to calving than primiparous. Dry buffaloes had 88.2% (OR = 0.118) lesser odds of abortion and six times (OR = 6.012) more likely to give birth than those lactating. The sex of the fetus was not a risk factor regarding abortion or calving. Other variables significantly associated with abortion rate were glucose and copper in the sera of aborted buffaloes were significantly higher (*p* < 0.05), and those of urea, uric acid, total protein, total cholesterol, phosphorus, magnesium and iron were significantly (*p* < 0.05) lower than a normal pregnancy. In conclusion, the present results emphasize that the identification of the risk factors, serum minerals and metabolites associated with fetus abortion of Egyptian buffalo may provide useful information, which assists to construct suitable preventive measures to raise reproductive performance.

## 1. Introduction

The buffalo (*Bubalus bubalis*) holds great promise for animal production in many countries [1]. However, buffaloes are characterized as a poor breeder in general because of late maturity, seasonal breeding, long postpartum anestrous and calving interval, poor expression of estrus and sub-optimal conception rate [2], which may further be complicated by abortions [3]. There are several factors involved in fetal loss from abortion, such as management, nutrition, as well as congenital and environmental factors [4]. Abortion in buffaloes is an important cause of production losses and low profitability in livestock farms [5]. On the other hand, late abortion decreases the number of calves available for sale and also reduces the potential number of replacement heifers [6]. Abortion is defined as the premature expulsion of the fetus from the dam between 45–300 days of gestation and usually occurs because the fetus has died in-utero [7]. The abortion rate of 3–5 abortions per 100 pregnancies per year on an animal farm is often considered normal, but if the fetal loss is more than 3–5% per year, then it becomes a matter of concern for the owner as well as veterinarians [3]. In buffaloes, the rate of abortion was found to reach the rate of 7% in Murrah buffaloes [4,8]. Meanwhile, it was 1.33% in Egyptian buffalo [9]. The causes of abortion are multi-factorial and can be non-infectious or infectious. Infectious causes of abortion associated with abortion in buffaloes include bacteria, viruses, protozoa and fungi [10]. Non-infectious maternal and paternal factors that cause abortion such as parity, sire effect and age at conception [11,12]. Genetic and non-genetic disorders could be some of the non-infectious factors that cause abortion [13]. Many infectious agents have been associated with abortion, although little is known about the multicausality of abortion, particularly with respect to trace elements. Associations between dam elements deficiencies and abnormal fetal development, including fetal loss from abortion, have been studied [14].

The use of serum metabolic profiling is a significant test in herd health assessment [15]. Animal nutritional status can be evaluated using the analysis of blood biochemical parameters [16]. Irregularities in numerous biochemical factors have been held liable for reproductive disorders in animals [17].

Although all causative agents have not the same potential to cause abortion in buffaloes, restricted reviews are available in this field, which needs further investigations. The identification of risk factors associated with abortion that could aid in controlling abortion and preventing this economic loss is vital for a breeder in Egypt. Due to the limited studies on risk factors affecting fetus abortion in the Egyptian buffaloes using multivariate logistic regression analyses, the present study was undertaken to investigate how potential risk factors (BCS, parity, season, lactation and sex), as well as certain serum minerals and metabolites, affect fetus abortion and calving rates of the Egyptian buffaloes in order to create the applicable control technique of abortion and develop the management system of herd health.

## 2. Materials and Methods

### 2.1. Data and Location

The data used to conduct this study were collected over seven consecutive years from 2014 to 2020 from 1154 records obtained from 364 Egyptian buffaloes (*Bubalus bubalis*), belonging to three private farms located in Al Sharkia governorate (El-Shorouk, El-Taaween, and El-Omda), Lower Egypt. Al Sharkia has a desert climate, and the average temperature in summer is about 27 °C, and in winter, it is 18 °C. A list of the all raw data containing number of animals, season, BCS, parity, age and number of abortion is provided in Supplementary Table S1

### 2.2. Animals and Management

Buffaloes were clinically healthy with average age, 3–15 years; weight 350–450 kg; average milk production is 8 kg/day. In order to assess the nutritional status of each buffalo, BCS was evaluated by using a 5-point [18]. Animals were milked twice daily (at 6 a.m. and 6 p.m.) with hand milking. Buffaloes were vaccinated for blackleg, foot and mouth disease and Anthrax. Young calves were vaccinated by *B. abortus* strain 19 and recently by Rb51. Additionally, herds were tested for Brucellosis and tuberculosis on a quarterly basis. Buffaloes were housed in the same shed, where 50% of the yard area was sheltered, and the animals had free access to open air. Each animal was identified by an ear tag. Reproduction of the herd was basically by natural mating. Animals fed on forage dry matter (Egyptian clover, alfalfa) together with a concentrate mixture (1.5 kg per animal for body maintenance, and extra concentrate of 0.5 kg/buffalo was fed 2–3 weeks before the expected date of calving) with free access to water. The concentrate mixture was composed of 30% barley, 21% yellow corn, 20% soybean meal, 25% wheat bran, 2% di-calcium phosphate, 1% common salt and 1% premix. Wheat straw was available ad libitum. These rations provided 12% crude protein (CP) and 67% total digestible nutrient (TDN).

### 2.3. Pregnancy Diagnosis

Pregnancy diagnosis was performed between 45 (placenta would be completed, and the fetus is dependent on its dam at this period) and 60 days of gestation and then repeated approximately 2–3 months before expected calving by palpating per rectum the uterus and its contents.

### 2.4. Clinical Diseases and Serology Tests

The buffaloes were determined to be clinically healthy upon physical examination and BCS. In addition, blood samples from aborted buffaloes were collected to investigate buffalo’s abortion (sero-diagnosis). The sera samples were divided for *Brucella abortus* antibodies, the Rose Bengal test was applied, and the antigen (Rose Bengal-stained antigen) was obtained by VSVRI (Abbasia Laboratories, Cairo, Egypt). Serological testing against BVD was performed also using antigen-capture ELISA of commercial ELISA bovine viral diarrhea antigen mix screening kit (Institut Pourquier®, Montpellier, France). The detection of antibodies against *Neospora caninum* was performed also by using *N. caninum* agglutination test (NAT). To identify *T. fetus* mucus and vaginal washing fluids were used for in vitro cultivation in Diamond’s medium (Remel, San Diego, CA, USA). We registered buffaloes found to suffer clinical diseases or seropositive to infectious diseases and withdrew them from the study. Thus, we included only buffaloes considered to be in good health and aborted due to non-infectious causes in this study.

### 2.5. Abortion and Age of Aborted Fetus Traits

Abortion was considered if any of the following events arisen between 45 and 300 days of gestation (after pregnancy diagnosis), buffaloes that expelled fetus or fetal membrane; or returned to service after being definitely pregnant; or in pregnant buffaloes that were found open during reconfirmation of pregnancy at the time of dry-off. The aborted fetal age was estimated to the nearest month using the information on uterine size and involution and dates of estrus, palpation to diagnose pregnancy and the curved crown-rump length (CVR) measured to the nearest centimeter by applying previously established formulas. For fetuses with a CVR length calculating less than 20 cm. the age was calculated according to the prescription: Y = 28.660 + 4.496x, where Y is the age in days and x the CVR length in centimeters. The prescription: Y = 73.544 +2.256x was pragmatic for computing the age of fetuses of a CVR length of 20 cm. or more [19]. Considered aborted fetuses through the trimesters of gestation were divided into three groups as follows: first (<4 months), second (4–7 months) and third (>7 months).

### 2.6. Blood Sampling and Analysis Protocol

Blood samples were obtained aseptically by jugular venipuncture [20,21]. The blood samples were collected from all observable aborted buffaloes (brucellosis and tuberculosis free, and BVD, *T. fetus* and *N. caninum seronegative*) during the first trimester (non-pregnant on a follow-up examination) and from normal pregnant animals of the same gestational age, separately. A total of 70 blood samples were collected (35 aborted buffaloes and 35 normal pregnant buffaloes). Each blood sample was centrifuged at 3000 rounds per minute (rpm) for 15 min [22]. The serum was separated and kept at −20 °C until the time of analysis.

### 2.7. Biochemical Analysis

Analysis of glucose, urea, creatinine, uric acid, total proteins, total cholesterol, phosphorus (P), magnesium (Mg), iron (Fe), copper (Cu) and zinc (Zn) concentrations. Glucose (mg/dL) was determined by using an enzymatic colorimetric method using the Bio-Diagnostic^®^ kit, Egypt, according to and conducted by a semi-automated clinical chemistry analyzer Photometer 5010 V5+ (ROBERT RIELE GmbH & Co. KG, Berlin, Germany) [23,24,25,26,27].

### 2.8. Statistical Analyses

For each reproductive event included abortion or calving rates, a multivariate mixed-effects logistic regression model was run throughout PROC logistic [28] in order to examine their relationships with the potential risk factors. The statistical model was incorporated as follows.

[Log (ΠABR/(1 − ΠABR)) = β0 + β1F + β2 BCS + β3 P + β4SE + β5L+ β6S + β7Y]

where ΠABR is the probability of abortion or calving, F is farm effect, BCS is body condition score, P is dam parity, SE is calving season, L is lactating, S is aborted fetus sex, and Y is calving year.

BCS, parity, season, year, lactation and sex were included in the statistical model as fixed effects, while the farm was considered as a random effect. The aborting year was included in the model to control for its effects on dependent variables. The odds ratio (OR) is a statistical assessment that considered the strength of the association between two events. We used this analysis as an approximate measure of relative risk to clarify the association effects of some risk factors on abortion and calving rates. Confidence intervals (95%) for OR estimates [29]. Calving rates were calculated as the number of buffalos for which the calving took place divided by the number of pregnant buffalos. Pregnancy rates were calculated as the number of pregnant buffalos (calving and aborting animals) divided on the number of buffalos naturally mated. The abortion rate was calculated as the number of observed aborting buffalos divided by the number of pregnant buffalos (aborted plus calving ones). The differences in biochemical blood parameters between aborted and normal cases were detected by a student’s t-test. Sample size was detected at 95% (CI) = 1.96 [30]. A *p*-value of <0.05 was considered statistically significant.

## 3. Results

### 3.1. Overall Distribution of Pregnancy, Abortion, and Survival Rates

Data presented in Table 1 showed the distribution of pregnancy, abortion, and survival rates. Abortion accounted for 5.89%, and the percentage of calving rates was 94.11%. The highest incidence of abortion in buffaloes occurred within the first trimester, with 3.03% of all cases in this study, followed by the second trimester 1.73% and the lowest in the third trimester 1.13%.

### 3.2. Potential Risk Factors

Table 2 and Table 3 illustrated the logistic regression analysis of risk factors associated with abortion rates and, therefore, calving rates in Egyptian buffalo. There were significant effects (*p* < 0.05) of body condition score (BCS), parity, season, and lactation on aborting and calving rates, while aborted fetus sex and aborting year had no significant effects (*p* > 0.05). Compared to the thinnest buffalos, average, fat and obese animals were 42.1%, 75.1% and 73.2%, respectively, less likely to abort (OR = 0.579, 0.249 and 0.268, respectively). Parity was another factor that affected aborting rates. Multiparous buffaloes were less likely to abort than primiparous; the aborting probability decreased to 59.1% and 67.7% (OR = 0.409 and 0.323) for those in fourth and fifth parities, respectively. In comparison with the spring season, aborting probability decreased 49.7% (OR = 0.503) during the winter season, while the animals were 85% (OR = 1.855) and 31% (OR = 1.316) more likely to abort in the summer and autumn seasons, respectively. Interestingly, dry buffaloes were 88.2% (OR = 0.118) less likely to abort than lactating (Table 2). With regard to calving rate, our results showed that the animals with body energy reserves (fat and obese) were 3.734 and 4.014-times, respectively, more likely to calving than the thinnest. In comparison to the animals in the first parity, the animals in the fourth and fifth parities had a 2.447 and 3.098-times, respectively, higher chance of calving. The season was another factor related to calving rates, calving probability increased 72.1% (OR = 1.721) during the winter season, while the animals were 53.1% (OR = 0.469) and 24% (OR = 0.760) less likely to calving in summer and autumn seasons, respectively, than those in the spring season. Dry buffaloes had six times (OR = 6.012) higher chance of calving than lactating (Table 3).

### 3.3. Biochemical Blood Parameters

The means, along with their standard errors of various biochemical parameters related to abortion rates of aborted and control animals, are shown in Table 4. The levels of glucose and copper in the sera of aborted buffaloes were significantly higher (*p* < 0.05), and those of urea, uric acid, total protein, total cholesterol, phosphorus, magnesium, and iron were significantly lower (*p* < 0.05) than normal pregnant controls. Meanwhile, non-significant differences (*p* > 0.05) were detected between aborted buffaloes and normal pregnant controls in creatinine and zinc.

## 4. Discussion

In the present study, the overall distribution of abortion and survival rates (5.89% and 94.11%, respectively) was reported. The abortion rate reported in our study is slightly higher than that found by Afifi et al. (4.1%) [31]. In addition, it is higher than the 1.51% reported by Ahmed and Zaher [32] in Egyptian buffaloes and lower than that found by Khan et al. [5], who noticed that the abortion and survival rates in Murrah buffaloes were 7.1% and 92.89% respectively. The differences in abortion and survival rates between studies may be attributed to the differences in the study population and the management characteristics between farms and spread of infection during some years, whether it is a bacterial, viral or fungal infection which would raise abortion rates.

The highest incidence of pregnancy loss in buffaloes occurred within the first trimester, with 3.3% of all cases in this study. This agrees with the results of Deresa et al. [33] in cows and Vecchio et al. [34] in buffaloes, who found that there was a significantly (*p* < 0.01) higher incidence of abortion during 28–60 days of gestation and lower after 71 days of gestation. Those authors suggest that the higher incidence of abortion could be due to the lower levels of serum progesterone that normally found in the transitional period (from 3 weeks to 3 weeks after calving) in buffalo cows.

An interesting finding noted in this study, the risk of abortion in thin buffaloes was higher. These results are similar to those reported by Mellado et al. [35] in goats, who found the thinnest goats were nine times more likely to abort. This result is consistent with the findings of other reports that show cows in poor BCS are more likely to abort [36,37].

Primiparous buffaloes had a higher risk of abortion than multiparous buffaloes. Similar results were recorded by Lu et al. [38], Grimard et al. [39], Al-Samarai [40] and Hashmi et al. [8]. The higher abortion rate in primiparous buffaloes may be explained because of hormonal disturbance and abnormal maternal recognition, the greater nutrient demands in these categories of animals to achieve mature body size and the negative energy balance during this period, as well as the reduction in body condition score, which would be harmful to fetal survival. Primiparous buffaloes may have less acquired immunity and may be more susceptible to infectious agents than are multiparous [7]. According to El-Regalaty [9], mature animals have a greater larger pelvic area and body size compared to immature animals; therefore, adult animals are capable of developing and giving birth to healthier calves. The first parity buffaloes had a higher rate of abortion than multiparous buffaloes. However, Segura-Correa [6] found that second parity cows had a higher risk of abortion followed by the third and first parity cows, as compared to multiparous cows. Bhat et al. [41] concluded that the abortion rate in dairy cattle at first parity was 48.96% and began to decrease with the progress of dam parity being 3.73% at the fifth parity.

The higher incidence of abortion during the summer season was recorded in this study. This might be due to higher temperature and humidity are more likely to affect the spread of infectious agents, and heat stress may also result in abortion. Other reasons that increase the probability of abortions during this season include an insufficient secretion of progesterone [8]. This finding is in agreement with the previous results of Bhat et al. [41], who found that the risk of abortion in the summer season was higher than in other seasons. However, El-Regalaty [9] reported that the abortion rate during the cold season was significantly (*p* < 0.05) doubled that recorded during the hot season (68.8% vs. 31.2%). Mohanty et al. [4] stated that calving season had no significant effect on the incidence of reproductive disorders, including abortion and stillbirth. The observed difference in the reports could be attributed to the variation in environmental conditions and system of management.

The incidence of abortion in lactating buffaloes was higher than that of dry buffaloes, with a significant difference like in the other report [42] in cows. The higher rates of abortion in lactating buffalo compared to dry buffaloes may be attributed to the negative effects of metabolic demands of lactation on fetal development.

The sex of the calf was not an important risk factor on abortion, as have been reported by Roche et al. [43] and Al-Samarai [40]. However, Bhat et al. [41] reported that the abortion rates in the male fetus are higher than abortions in female ones (61.41% vs. 38.6%).

Regarding the biochemical results, a significant increase in glucose concentration was detected in aborted buffaloes. Similar findings were recorded by Giri et al. [44] in cows, and they explained the hyperglycemic response in the aborting animals may be due to glycogenolysis and gluconeogenesis due to increased concentrations of catecholamines [45] and cortisol [44], respectively. On the other hand, the low level of glucose in the present study was observed in normal pregnant buffaloes may be due to developing fetuses and mobilization of maternal glucose for providing the growing fetus with energy [46].

Concerning the total protein level in the present study, there was a significant decrease in the serum levels of total protein in aborted buffaloes compared with the healthy control. These results were in line with the previous findings that hypoprotenemia was due to liver damage caused by chronic parasitism, which leads to a decrease in albumin synthesis in the liver [47]. The decrease in the serum total protein concentration observed in aborted animals might be attributed to the restricted food intake in aborted buffaloes.

Concerning the serum creatinine level, the aborted buffaloes showed a non-significant change as compared with the normal control. Meanwhile, the serum urea concentration was significantly decreased in aborted buffaloes. The reduced serum urea levels in the aborted buffaloes may be due to the damage of hepatic tissue, which cannot form urea from ammonia [48] or the use of urea for protein synthesis on the rumen-hepatic pathway due to compensation of the low protein uptake [49]. This result disagrees with the results of Rhoads et al. [50] and Molefe and Mwanza [51], who found significantly higher urea mean concentrations in aborting cows. Previous studies suggest that a higher urea results in decreased reproductive performance as a consequence of the combined effects of changes in uterine pH [52], increased uterine luminal PGF2α [53] and reduced plasma progesterone concentration in lactating animals [54].

This study was up to look at the association between oxidative stress and abortion in buffaloes. From the study, we found that the uric acid concentrations, as an indicator of total antioxidant status, revealed significantly lower values in the aborted buffaloes. This finding agrees with previous works reported; uric acid serves as a potent antioxidant by radical scavenging, and a decrease in uric acid is associated with low protein uptake and is useful in the diagnosis of pathological conditions of the late pregnancy [55] and disagrees with the results of Khalaf et al. [56] in human. The decrease in the uric acid levels during the time of abortion suggests that the uric acid is used at the time of abortion as an antioxidant.

Cholesterol mean concentrations of aborting buffaloes were significantly lower in the present study. Similar results were recorded by Molefe and Mwanza [51] in cows. Negative energy balance, poor body condition and low Cholesterol concentrations are usually indicators for health illnesses and poor nutrition status in cows [57], which could explain the low level of CHOL in aborting buffaloes.

A significantly lower magnesium concentration was seen in aborting buffaloes in this study. These results are similar to those reported by Molefe and Mwanza and Lanyasunya et al. [51,58] in aborting cows and Mellado et al. [35] in goats. Lower maternal (Mg) concentrations are associated with abortion by inducing uterine hyperexcitability and overactivity, ending with abortion or preterm

In this study, it was found that serum Cu levels in aborted buffaloes were higher than the normal pregnant. These results were in agreement with earlier results by Ekin et al. [59] in cows and Aytekin and Aypak [60] in sheep but were different from those reported by Barui et al. [61]; Regmi1 and Dhakal [62] and Modi et al. [63] in cows. It is reported that the highest increases in copper levels are seen during acute and chronic infections [64].

Micronutrient minerals, especially iron, are very important during pregnancy when the developing fetus is very vulnerable to inappropriate micronutrient status [65]. Further, it is the most important element in blood, which contributes to hemoglobin concentration. In the present study, the concentration of iron obtained in the serum of aborted buffaloes was significantly lower as compared with normal pregnancies. These results were in agreement with earlier results by Modi et al. and Kumar et al. [63,66]. The lower phosphorus concentrations in aborted buffalo in the current study may be due to poor dietary intake and abortion stress [1].

The growing fetus requires Zinc for normal development [67]. In addition, zinc deficiency results in fetal anomalies and stillbirth [68]. Additionally, Harvery et al. [69] reported that (Zn) levels in porcine fetuses were higher than those in the endometrium, which further implies that fetuses have a capacity to sequester dam (Zn). In the current study, serum (Zn) levels in normal buffaloes were lower than the aborted are in good concurrence with previous findings by Oetzel [15], which implies an efficient mechanism of increased dam (Zn) utilization by the developing fetus.

## 5. Conclusions

Body condition score, season, parity number and lactation were significant risk factors that result in the high abortion rate in buffaloes. The highest incidence of abortion occurred during the first trimester of gestation. The differences in serum minerals and metabolites levels were seen in aborted buffaloes. Particularly, variations of serum levels glucose, magnesium and copper in the sera of aborted buffaloes were significantly higher, and those of urea, uric acid, total protein, total cholesterol, phosphorus and iron were significantly lower. The result reported in this study serves as a useful tool to create the applicable control technique of abortion.

## Figures and Tables

**Table 1:** The prevalence of pregnancy, occurrence of abortion and survival rates in three private farms in Al-Sharkia.

No. of Confirmed Pregnant Buffaloes	No. of Aborted Fetuses (%)	Gestational Age of Aborted Fetus *			Survival Rate (%)
		First Trimester (%)	Second Trimester (%)	Third Trimester (%)	
1154	68/1154 (5.89)	(35/1154) 3.03	(20/1154) 1.73	(13/1154) 1.13	1086/1154 (94.11)

* Trimesters of gestation were the first <4 months, second (4–7 month) and third >7 month, respectively.

**Table 2:** Logistic regression analysis of risk factors associated with fetus abortion of Egyptian buffalos.

Items	*n*	β	S.E.(β)	Abortion Rate		*p*-Value
				OR	95% CI	
**Body Condition Score**						
2–2.5	425			1		
3–3.5	488	−0.546	0.485	0.579	0.224–1.499	0.001
4–4.5	151	−1.316	0.320	0.268	0.143–0.503	0.001
5	90	−1.390	0.529	0.249	0.088–0.702	0.001
**Parity**						
1st	246			1		
2nd	240	−0.188	0.330	0.828	0.433–1.583	0.062
3rd	283	−0.417	0.394	0.659	0.304–1.427	0.012
4th	180	−0.894	0.368	0.409	0.198–0.842	0.001
5th	205	−1.130	0.470	0.323	0.128–0.811	0.001
**Season**						
Spring	227			1		
Summer	247	0.456	0.339	1.855	0.940–2.663	0.006
Autumn	395	0.274	0.369	1.316	0.638–2.714	0.048
Winter	285	−0.686	0.436	0.503	0.214–1.185	0.002
**Lactation**						
Lactating	623			1		
Dry	531	−2.140	0.240	0.118	0.073–0.189	0.001
**Aborted fetus sex**						
Male	603			1		
Female	551	0.099	0.136	1.105	0.846–1.444	0.622
**Aborting year**						
2014	140			1		
2015	129	−0.651	0.238	0.739	0.348–1.328	0.098
2016	179	−0.462	0.294	0.775	0.307–1.576	0.180
2017	173	−0.683	0.363	0.692	0.231–1.180	0.089
2018	213	−0.363	0.116	0.987	0.434–2.246	0.261
2019	142	−0.436	0.256	0.972	0.446–2.120	0.236
2020	178	−0.534	0.317	0.754	0.324–1.634	0.117

β, Regression coefficient; S.E.(β)., Standard error; OR, Odds ratio.

**Table 3:** Logistic regression analysis of risk factors associated with calving rates of Egyptian buffaloes.

Items	*n*	β	S.E.(β)	Calving Rate		*p*-Value
				OR	95% CI	
**Body Condition Score**						
2–2.5	425			1		
3–3.5	488	0.631	0.286	1.728	0.667–4.472	0.032
4–4.5	151	1.293	0.227	3.732	1.990–4.998	0.001
5	90	1.483	0.431	4.017	1.424–7.337	0.001
**Parity**						
1st	246			1		
2nd	240	0.119	0.293	1.207	0.632–2.307	0.096
3rd	283	0.439	0.263	1.517	0.701–2.286	0.038
4th	180	0.778	0.332	2.447	1.188–4.040	0.001
5th	205	1.155	0.424	3.098	1.233–5.785	0.001
**Season**						
Spring	227			1		
Summer	247	−0.532	0.396	0.469	0.191–1.173	0.016
Autumn	395	−0.223	0.411	0.760	0.368–1.567	0.068
Winter	285	0.586	0.583	1.721	0.844–3.677	0.002
**Lactation**						
Lactating	623			1		
Dry	531	1.834	0.310	6.012	5.303–7.632	0.001
**Aborted fetus sex**						
Male	603			1		
Female	551	−0.085	0.101	0.905	0.692–1.183	0.364
**Aborting year**						
2014	140			1		
2015	129	0.416	0.203	1.339	0.751–1.685	0.073
2016	179	0.227	0.259	1.114	0.658–1.685	0.135
2017	173	0.448	0.328	1.438	0.634–2.260	0.064
2018	213	0.128	0.081	1.013	0.445–2.307	0.216
2019	142	0.201	0.221	1.029	0.472–2.245	0.211
2020	178	0.299	0.282	1.217	0.848–2.136	0.082

β, Regression coefficient; S.E.(β)., Standard error; OR, Odds ratio.

**Table 4:** A comparison of the mean values of various blood biochemical parameters in buffaloes presenting abortion and normal pregnant.

Parameters	Normal Cases	Abortion Cases	*p*-Value
Glucose (mg/dL)	28 *±* 3.128	61 ± 6.037	0.003
Urea (mg/dL)	46.5 *±* 1.362	34.5 *±* 1.119	0.035
Creatinine (mg/dL)	1.46 *±* 0.214	1.64 *±* 0.152	0.437
Uric acid (mg/dL)	2.4 ± 0.024	1.2 ± 0.018	0.029
Total protein (g/dL)	8.4 ± 0.027	6.8 ± 0.012	0.041
Total cholesterol (mg/dL)	98 ± 3.634	58 ± 2.934	0.016
Phosphorus (mg/dL)	3.16 ± 0.017	2.64 ± 0.006	0.011
Magnesium (mg/dL)	3.72 ± 0.027	2.91 ± 0.017	0.037
Iron (mg/dL)	17.2 ± 1.224	6.3 ± 0.367	0.005
Copper (mg/dL)	57.1 ± 1.342	85.3 ± 2.012	0.043
Zinc (mg/dL)	69 ± 2.114	72 ± 2.625	0.421

## Data Availability

The data presented in this study are available on request from the corresponding author.

## Supplementary Materials

The following are available online at https://www.mdpi.com/article/10.3390/ani11072043/s1, Table S1: A list of the all raw data containing number of animals, season, BCS, parity, age and number of abortion is provided in supplementary table S1.
